# Protective Effects of Berberine-Loaded Black Seed (*Nigella sativa* L.) Oil Nanoemulsion in the Atherosclerotic Mouse Model

**DOI:** 10.5812/ijpr-162867

**Published:** 2025-12-06

**Authors:** Nasim Darjani, Negar Panahi, Pejman Mortazavi, Mohammad Kazem Koohi

**Affiliations:** 1 Department of Veterinary Basic Sciences, SR.C., ‎Islamic Azad University, ‎Tehran, Iran; 2 Department of Veterinary Pathobiology, SR.C., ‎Islamic Azad University, Tehran, Iran; 3 Department of Comparative Biosciences, Faculty of Veterinary Medicine, University of Tehran, ‎Tehran, Iran

**Keywords:** Atherosclerosis, Antioxidants, Berberine, *Nigella sativa* seed, Nanoemulsion

## Abstract

**Background:**

This study investigated atherosclerosis (AS), a pivotal contributor to the onset of coronary artery disease and other cardiovascular ailments.‎

**Objectives:**

The presents study examined a novel therapeutic strategy combining black seed oil [*Nigella sativa* L. (NS)] and berberine (BBR) in a C57BL/6J mouse model of AS.

**Methods:**

After preparing the NS nanoemulsion, gas chromatography-mass spectrometry (GC-MS) was used for analysis. A BBR-loaded black seed oil-based nanoemulsion (BBS) was developed and evaluated for its thermodynamic stability, viscosity, particle size, and dynamic light scattering (DLS) properties. Forty-eight male C57BL/6J mice (8 weeks old, 18 - 20 g) were divided into six groups and fed a modified AIN-76 diet with 25% dietary fat for 16 weeks to induce AS. Treatment began after 8 weeks through oral gavage for the remaining 8 weeks. Levels of low-density lipoprotein (LDL), high-density lipoprotein (HDL), triglyceride (TG), and total cholesterol were measured, along with the atherogenic coefficient and cardiac risk ratio. We also quantified malondialdehyde (MDA), superoxide dismutase (SOD), glutathione peroxidase (GPX), and catalase (CAT) activities, and assessed pathological changes in cardiac and aortic tissues using hematoxylin and eosin (H&E) staining.

**Results:**

The GC-MS analysis confirmed that NS oil met quality benchmarks, displaying stability with a zeta potential of -18.4 mV. The study showed a Polydispersity Index (PDI) of 0.3019 and a Z-average of 250.4 nm. The BBS improved lipid profiles in mice, increasing HDL and decreasing LDL, TG, and total cholesterol, thereby reducing cardiac risk. The formulation exhibited strong antioxidant effects, with reduced MDA levels and enhanced SOD, GPX, and CAT activities. Pathological observations supported these biochemical results.

**Conclusions:**

Our findings suggest that BBS offers valuable insights into the mechanisms underlying atherosclerotic disease. This understanding could pave the way for novel approaches to cardiovascular health and the development of effective preventive strategies.

## 1. Background

Atherosclerosis (AS), a major type of cardiovascular disease, is a chronic condition often culminating in serious cardiovascular incidents (1). The pathogenesis of AS involves the accumulation of atheromatous plaques, which are lipid-rich lesions composed of cholesterol, inflammatory cells, and fibrous tissue (2). When AS progresses, plaques enlarge and increasingly narrow the vascular lumen, impeding hemodynamic flow (3). The advancement of AS can culminate in primary coronary heart disease, often marked by coronary artery stenosis or severe vascular damage (4), characterized by intimal thickening, lipid deposition, and chronic inflammatory infiltration (5). Oxidized low-density lipoprotein (LDL) promotes AS via several mechanisms, including its cytotoxic properties, the induction of hypertriglyceridemia, and the facilitated transfer of triglyceride (TG) from VLDL and LDL to other cholesterol-enriched lipoproteins, such as LDL itself (6).

Herbal compounds are increasingly recognized as potential alternative or complementary therapeutic agents for treating a range of conditions (7). Recent research highlights the extensive medicinal efficacy of black seed, documenting various pharmacological actions attributed to black seed, including antioxidant, anti-inflammatory, anticancer, hepatoprotective, and immunomodulatory effects, along with demonstrated benefits in addressing male infertility, providing cardiovascular protection, enhancing memory, and exhibiting both antibacterial and antiviral activities (8).

Recent investigations underscore the capacity of berberine (BBR) to inhibit vascular smooth muscle cell proliferation, a key factor in mitigating vascular disorders (9). These observations suggest that BBR may have a significant impact on approaches designed to improve cardiovascular well-being. The BBR, the principal alkaloid extracted from Coptis chinensis, has a well-established history in traditional medicine, particularly recognized for its potent antimicrobial efficacy in managing dysentery and diarrhea (10). More recent research has expanded our understanding of BBR’s therapeutic potential, highlighting its antiarrhythmic effects and its capacity to induce vasodilation within the coronary vasculature (11).

An advanced drug delivery system (DDS) designed for targeted organ delivery aims to enhance therapeutic effectiveness, mitigate adverse effects related to blood lipids, and prevent the development of AS (12). In the pharmaceutical field, self-nano-emulsified drug delivery systems (SNEDDSs) are gaining traction as adaptable vehicles for various formulations, including creams, foams, liquids, and sprays (12).

This approach bolsters stability, thereby establishing oil-based nanoemulsions as proficient DDSs that augment the solubility and bioavailability of poorly water-soluble therapeutics (12).

## 2. Objectives

The present study aims to investigate the therapeutic efficacy of a BBR-loaded *Nigella sativa* L. (NS) oil nanoemulsion in an atherosclerotic murine model, thereby elucidating its potential role in enhancing cardiovascular disease treatment strategies. We hypothesized that the combined antioxidant and lipid-lowering effects of BBR and black ‎seed oil in a nanoemulsion (BBS) would synergistically attenuate AS progression in ‎C57BL/6J mice.‎

## 3. Methods

### 3.1. Collection and Storage of Nigella sativa Seeds

The NS seeds, acquired from a Tehran-based Iranian company, underwent a rigorous preparation process. The seeds were initially rinsed with both running and distilled water, followed by overnight air-drying in an oven set at 40°C. The dried material was then ground and sieved through a 250 µm mesh to yield a fine powder, which was then stored in amber vials under frozen conditions, awaiting further analysis.

### 3.2. Supercritical Fluid Extraction

A 150-gram powdered sample underwent processing using a Thar US Technology Supercritical Fluid Extraction (SFE)-1000F device (Pittsburgh, PA, USA). The extraction was conducted at 40°C and 60 MPa, with a CO_2_ flow rate of 20 mL/min, which is in contrast to a three-hour extraction performed at 25°C and 0.1 MPa, utilizing pure CO_2_ as the solvent (13).

### 3.3. Solvent Methods

Ten grams of nanoemulsion powder were accurately weighed and then dispersed into individual flasks, each containing 50 mL of solvent. For compound extraction, the flasks were sealed with aluminum foil and agitated at 700 rpm for four hours in a Heidolph Unimax 1010 shaking incubator. The initial extracts underwent a two-step filtration process utilizing Whatman No. 1 paper to eliminate particulate matter. Subsequently, a Heidolph Laborota 4000 rotary evaporator was employed to concentrate the extracts by removing the solvent. The resulting oil samples were then precisely weighed using a Shimadzu digital scale and stored at -80°C in anticipation of subsequent analytical procedures (13).

### 3.4. Gas Chromatography-Mass Spectrometry

Gas chromatography-mass spectrometry (GC-MS) analysis of the fixed NS oil was performed using a Shimadzu gas chromatograph interfaced with a QP2010 mass spectrometer. The specific composition of the oil was further characterized using a Thermo Finnigan TRACE GC-Polaris Q, equipped with a DB-Wax column (60 m × 0.25 mm ID × 0.25 µm film thickness). For each analysis, a 0.5 µL aliquot of the extracted oil was prepared by mixing it with a solvent blend of n-hexane and methanol (1:10 ratio), and subsequently injected in split mode at a ratio of 1:33 (13).

### 3.5. Synthesis of Black Seed Oil Nanoemulsion

A high-speed homogenization technique, with modifications, was employed to synthesize the nanoemulsion. Initially, 1% w/v BBR hydrochloride was dissolved in 10% v/v NS oil. The oil phase was then combined with surfactant solutions containing 10% v/v polyethylene glycol (PEG)-400 and 30% v/v Tween 80. The mixture underwent magnetic stirring at 600 rpm for 30 minutes, followed by homogenization at 10,000 rpm for 10 minutes to achieve optimal dispersion and stability (14).

### 3.6. Characterization of Black Seed Oil-Loaded Nanoemulsion

The emulsion sample was diluted by a factor of 100 with deionized water. A 1 mL aliquot of this diluted sample then underwent a further 100-fold dilution for zeta potential measurement using a Malvern Zetasizer Nano ZS. Viscosity measurements were performed in triplicate on undiluted samples at 25°C using a Brookfield LVF viscometer. Morphological analysis was conducted using a JEM-1200EX transmission electron microscope (TEM). Before imaging, the nanoemulsion was diluted to a 1:50 ratio, stained with 2% phosphotungstic acid, and then a drop was deposited onto a holey film grid (15).

### 3.7. Construction of Pseudo-Ternary Phase Diagram

A pseudo-ternary phase diagram was constructed at 24°C via a water titration method to delineate the concentration ranges conducive to nanoemulsion formation. Various surfactant system-to-oil volume ratios (1:9 to 9:1) were prepared using diverse surfactant system blend ratios (1:0, 1:1, 1:2, 2:1, 3:1, and 3:2). Water was incrementally added to these mixtures until turbidity indicated the phase boundary, at which point the quantities of each component were documented for diagram generation. The resulting nanoemulsions exhibited monophasic, transparent, low-viscosity, and stable characteristics (16).

### 3.8. Thermodynamic Stability

The thermodynamic stability of the enhanced formulation was assessed using a controlled cooling-heating cycle. The evaluation involved maintaining the formulation at 4°C for 48 hours, followed by storage at 48°C for an additional 48 hours. This heating-cooling sequence was repeated for a total of three cycles to determine the emulsion’s performance (17).

### 3.9. Stability Index

The stability of the optimized nanoemulsion was assessed through a rigorous three-cycle freeze-thaw protocol. Each cycle involved freezing at -20°C for 24 hours, followed by thawing at room temperature. Subsequently, the nanoemulsion was subjected to centrifugation to determine any phase separation, as documented in reference (18). Additionally, the Stability Index of the essential oil nanoemulsion was quantified using: Stability Index = 100 × [original globule size (original globule size-changing globule size)/original globule size] (19).

To evaluate the emulsion’s stability, the nanoemulsion was subjected to a one-month storage period at 40°C. For optimal stability, the nanoemulsion should exhibit a droplet size ranging from 100 to 500 nm, possess a suitable viscosity, and maintain a negative zeta potential.

### 3.10. Animals

Forty-eight adult male C57BL/6J mice (age = 8 weeks; weight = 18 - 20 g) were procured from the Pasteur Institute of Iran (Tehran, Iran)‎ and maintained in accordance with the university’s ethical guidelines. The mice were housed under controlled environmental conditions, including a 12-hour light/dark cycle, a temperature range of 23 - 25°C, and 55% humidity. After a seven-day acclimatization, mice were randomly divided into six groups of eight (n = 8) based on previous studies using atherosclerotic models. The C57BL/6J strain is notably sensitive to chronic oral gavage stress, with mortality rates of 12 - 18% in long-term studies. This sample size accounts for their vulnerability to stress, ensuring robust statistical power despite potential attrition.

### 3.11. Induction of an Atherosclerosis Mouse Model

The AS was induced in C57BL/6J mice through a modified AIN-76 diet. The 25% dietary fat was part of the modified AIN-76 diet (20). This modification involved supplementing the diet with 1.25% (w/w) cholesterol, 0.5% (w/w) cholate, 5% egg yolk, 5% corn oil, and 13.75% cocoa oil (20). Concurrently, the mice were provided with 5% sucrose in their drinking water. ‎Experimental groups also received daily oral treatments from the study’s eighth week until its conclusion at 16 weeks, in conjunction with a diet containing 25% dietary fat. Prophylactic treatments were given starting in the 8th week to assess their preventive effects.

- Group 1: Healthy control mice (H) received physiologic saline

- Group 2: The atherosclerosis model (AS) received no treatment

- Group 3: The atherosclerosis model (SIM) received simvastatin (5 mg/kg and 0.1 mL) for 8 weeks

- Group 4: The atherosclerosis model (B) received BBR (1% and 0.1 mL) for 8 weeks

- Group 5: The atherosclerosis model (BS) received black seed oil-based nanoemulsion (0.1 mL) for 8 weeks

- Group 6: The atherosclerosis model (BBS) received BBR-loaded black seed oil-based self-nanoemulsion (0.1 mL) for 8 weeks. All mice completed the 16-week study protocol, and no animals were excluded from the final ‎analysis.‎

### 3.12. Blood Sampling

Blood was drawn from the animal’s heart and collected in plain tubes, then permitted to clot at room temperature. Subsequently, the samples underwent centrifugation at 3000 × g for 15 minutes to isolate the serum.

### 3.13. Tissue Preparation

Following a 16-week experimental period, the animals were anesthetized via intraperitoneal administration of a ketamine (90 mL) and xylazine (10 mL) mixture. Each mouse received 0.1 mL of the cocktail until an appropriate anesthetic plane was achieved. Subsequently, the cardiac and aortic arch tissues were collected and preserved in formalin for subsequent histological or molecular analyses.

### 3.14. Measurement of Serum Lipid Profiles

Following the isolation of mouse serum, high-density lipoprotein (HDL), LDL, TG, and total cholesterol concentrations were quantified. These measurements were performed via enzymatic assays using an automated biochemical analyzer (Model AD2700, Olympus, Tokyo, Japan). Additionally, the atherogenic coefficient and cardiac risk ratio were computed (21).

### 3.15. Oxidative Stress Analysis

Oxidative stress markers within liver tissue were quantitatively assessed. The specific markers investigated included glutathione peroxidase (GPX), superoxide dismutase (SOD), malondialdehyde (MDA), and catalase (CAT). Commercial assay kits for MDA and GPX were acquired from Kiazist, Iran, while SOD and CAT kits were sourced from Kushanzist, Iran. All measurements were performed using an enzyme-linked immunosorbent assay (ELISA) reader (DANA, Iran).

### 3.16. Histopathology

Aortic tissues underwent fixation in 10% buffered formalin. Subsequently, 5 µm tissue sections were stained using the hematoxylin and eosin (H&E) protocol. The thickness of the intima and media layers of the smooth muscle endothelium was assessed. The endothelial layer was classified into four grades: Zero for standard thickness, 1 for mild hyperplasia (0 - 25%), 2 for moderate hyperplasia (25 - 50%), and 3 for severe hyperplasia (50 - 75%) compared to the healthy group. The quantity and dimensions of lipid plaques were evaluated, with no plaque receiving a grade of 0, small plaques receiving a grade of 1, medium-sized plaques receiving a grade of 2, and large plaques receiving a grade of 3. To assess tissue inflammation, the percentage of foam cell and macrophage infiltration was evaluated, where grade 0 represents an absence of inflammatory cells, low macrophage infiltration (0 - 25%) is scored as grade 1, moderate macrophage infiltration (25 - 50%) is designated as grade 2, and high infiltration (50 - 75%) corresponds to grade 3 with the control group.

### 3.17. Statistical Analysis

‎A one-way ANOVA was used for statistical evaluation, followed by Tukey’s post hoc analysis ‎by GraphPad Prism version 10.4.1. The characterization of the BBS nanoemulsion ‎is presented ‎as Mean ± SD, while the lipid profile and oxidative stress markers are shown as ‎Mean ± SEM. ‎The Kruskal-Wallis test was used for statistical assessment to evaluate the ‎pathological score. A ‎P-value < 0.05 was considered statistically significant.

## 4. Results

### 4.1. Gas Chromatography-Mass Spectrometry Studies

The GC-MS analysis was conducted to assess the quality attributes of the extracted black cumin oil. The fatty acid analysis revealed several key components, including C16:0 (palmitic acid at 0.23%), C18:0 (stearic acid at 2.69%), C18:1(9) (oleic acid at 5.17%), C18:1(12) (petroselinic acid at 66.10%), 10-nonadecanone, C18:2(9,12) (linoleic acid at 18.16%), C18:3(9,12,15) (linolenic acid at 7.51%), and C20:0 (arachidic acid at 0.03%). The sample’s quality profile was further enhanced by the presence of Vitamin E (3.56 mg/100g) and a substantial concentration of total phenolic compounds [9.14 mg gallic acid equivalents (GAE)/g].

### 4.2. Characterization of Black Seed Oil Nanoemulsion

#### 4.2.1. Dynamic Light Scattering and Determination of Zeta Potential

**Figure 1.**
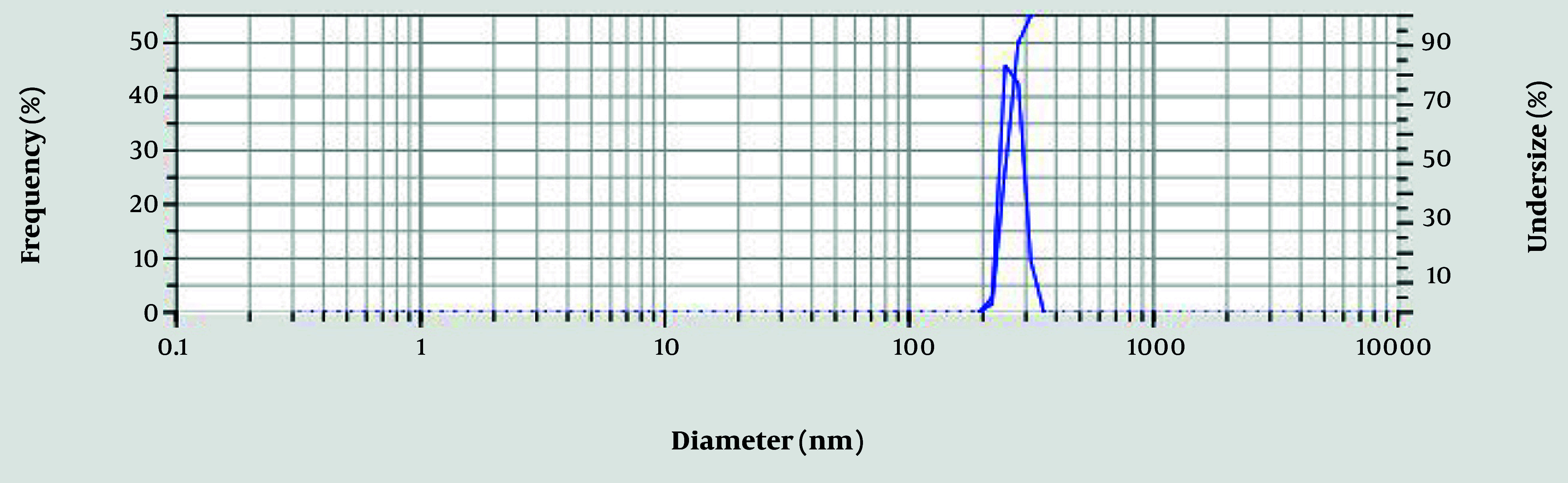
Characteristics of the size of nano-emulsion formulation using the dynamic light scattering (DLS) technique

**Figure 2.**
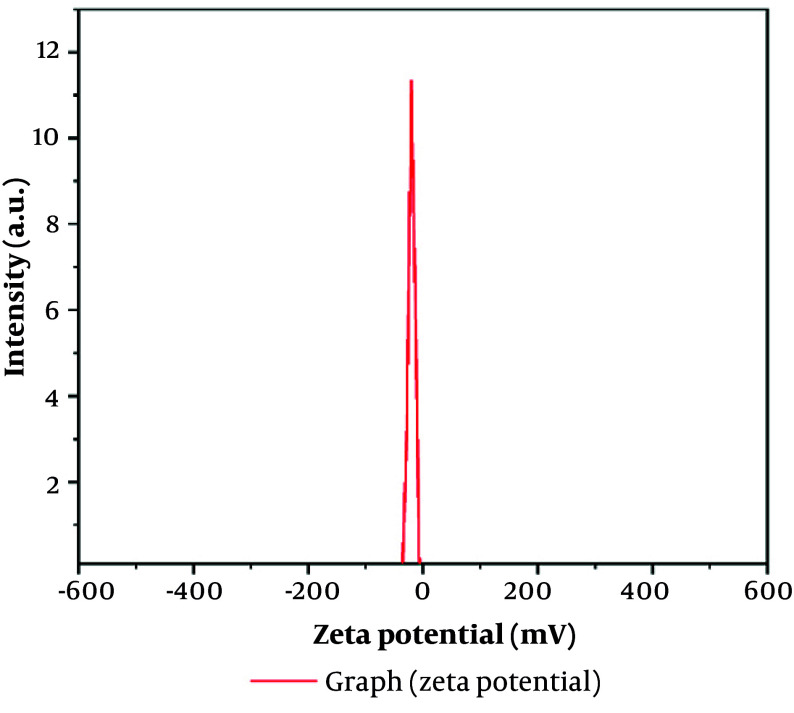
Zeta-potential distribution of nano-emulsion formulation using dynamic light scattering (DLS) technique (B).

The mean size of the nanoparticles is 250.4 nm, which is appropriate for BBS nanoemulsions. The particle size distribution was characterized by moderate polydispersity, with a Polydispersity Index (PDI) of 0.3019 (Figure 1) (22). Furthermore, a zeta potential of -18.4 mV was recorded (Figure 2 and Table 1), suggesting moderate colloidal stability.

**Table 1.** Zeta Potential and Electrophoretic Mobility of the Optimal Nanoemulsion Formulation

Electrophoretic Mobility	Zeta Potential	Peak No.
**0.000143 cm** ^**2**^ **/Vs**	-18.4 mv	1

#### 4.2.2. Transmission Electron Microscope Image Analyses

**Figure 3.**
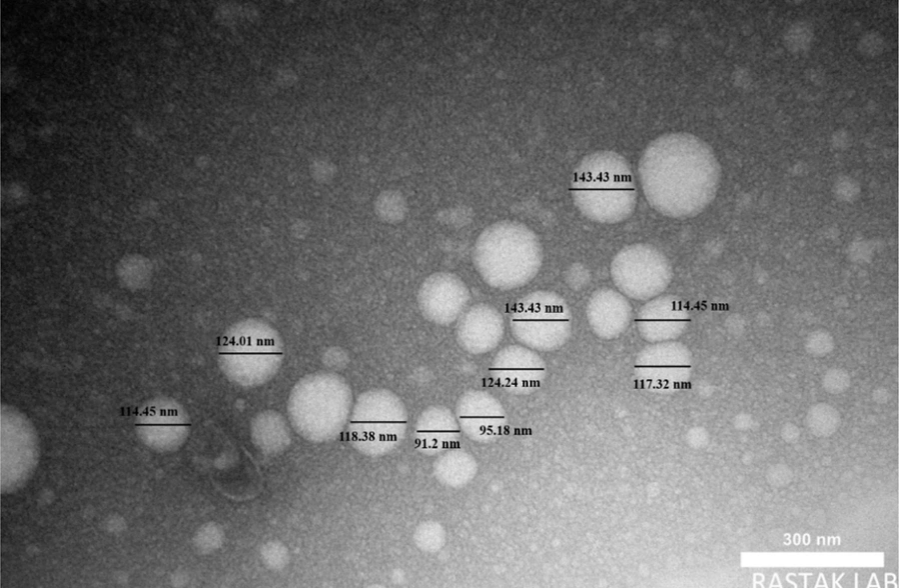
The transmission electron microscope (TEM) micrograph of the prepared nano-emulsion (scale 300 nm).

The TEM is a high-resolution imaging technique that provides direct measurements of the particle core’s actual size. This method specifically examines the solid structure, excluding any surrounding water layers or emulsifiers. The particle size, as determined from TEM images (Figure 3), was 117.32 nm.

### 4.3. Alterations in Serum Lipid Profiles

An analysis of serum lipid profiles in C57BL/6J mice demonstrated that treatment with SIM, B, BS, and BBS significantly elevated HDL levels compared to the AS group. Notably, the BBS treatment group exhibited a significantly greater increase in HDL than the SIM, B, and BS groups (P < 0.0001). In terms of LDL cholesterol level modifications, a statistically significant reduction was observed in the groups treated with H, SIM, B, BS, and BBS when compared to the AS group. Furthermore, the levels in the BS and BBS groups were notably lower than those in the B group (P < 0.0001). Interestingly, BBS levels demonstrated a significant decrease relative to B (P < 0.0001) yet an increase when compared to SIM (P < 0.01). The TG concentrations in the SIM, B, BS, and BBS groups showed a statistically significant reduction compared to the AS group (P < 0.0001). Conversely, these levels were significantly elevated compared to the H group (P < 0.0001). Furthermore, a notable increase in cholesterol levels was observed across the H, SIM, B, BS, and BBS groups in comparison to the AS group (P < 0.0001). Within the study, the BBS group demonstrated a significant decrease in cholesterol levels relative to both the B and BS groups (P < 0.0001, Table 2). 

**Table 2.** Effects of Nanoemulsion Berberine on C57BL/6 J Mice’s Serum Lipid Profiles ^a, b, c^

Groups	H	AS	SIM	B	BS	BBS
**HDL (mg/dL)**	33.05 ± 0.8 AB	26.93 ± 1.1 B	40.65 ± 1.9 CD	35.55 ± 0.59 AC	46.54 ± 2.44D	59.79 ± 2.03 E
**TG (mg/dL)**	76.38 ± 3.05 A	223.6 ± 4.83 B	136.1 ± 2.19 C	179.4 ± 3.1 D	158.8 ± 3.77 E	146.1 ± 3.44 EC
**Cholesterol (mg/dL)**	117.0 ± 3.6 A	363.6 ± 4.7 B	226.1 ± 5.6 C	293.9 ± 3.8 D	254.4 ± 4.2 E	217.3 ± 4.1
**LDL (mg/dL)**	58.50 ± ‎1.8A‎	776.8 ± ‎50.1B‎	208.8 ± ‎14.6‎C	623.1 ± ‎34.2D‎	313.9 ± ‎11.7E‎	318.8 ± ‎52.6E‎
**Atherogenic Coefficient**	2.54 ± 0.02 A	12.5 ± 0.75 B	4.56 ± 0.19 C	7.27 ± 0.42 D	4.46 ± 0.2 C	2.63 ± 0.02 A
**Cardiac Risk Ratio**	3.54 ± 0.02A	13.50.75 B	5.56 ± 0.19 C	8.26 ± 0.42 D	5.46 ± 0.19 C	3.63 ± 0.02 A

Abbreviations: HDL, high-density lipoprotein; TG, triglyceride; LDL, low-density lipoprotein.

^a^ H, healthy; AS, atherosclerotic model; SIM, AS and simvastatin (15 mg/kg); B, AS and berberine (30 mg/kg); BS, AS and nanoemulsion of black seed oil (10%); BBS, AS and berberine (1%) nanoemulsion of black seed oil (10%).

^b^ Values are expressed as mean ± SEM (n = 8).

^c^ A, B, C, D, and E indicate significant differences between groups.

### 4.4. Evaluation of Changes of Atherogenic Coefficient and Cardiac Risk Ratio

The analysis of the atherogenic coefficient showed a significant increase in the AS group compared to the H group (P < 0.001). In contrast, the SIM, BS, and BBS groups exhibited a notable reduction compared to the AS group (P < 0.01, P < 0.01, and P < 0.001, respectively). The analysis of cardiac risk ratios demonstrated a statistically significant increase in the AS group when compared to the H group (P < 0.01). Conversely, the SIM, BS, and BBS groups exhibited a significant reduction in cardiac risk ratios relative to the AS group (P < 0.05, P < 0.05, and P < 0.01, respectively, Table 2). 

### 4.5. Oxidative Stress Analysis

**Figure 4.**
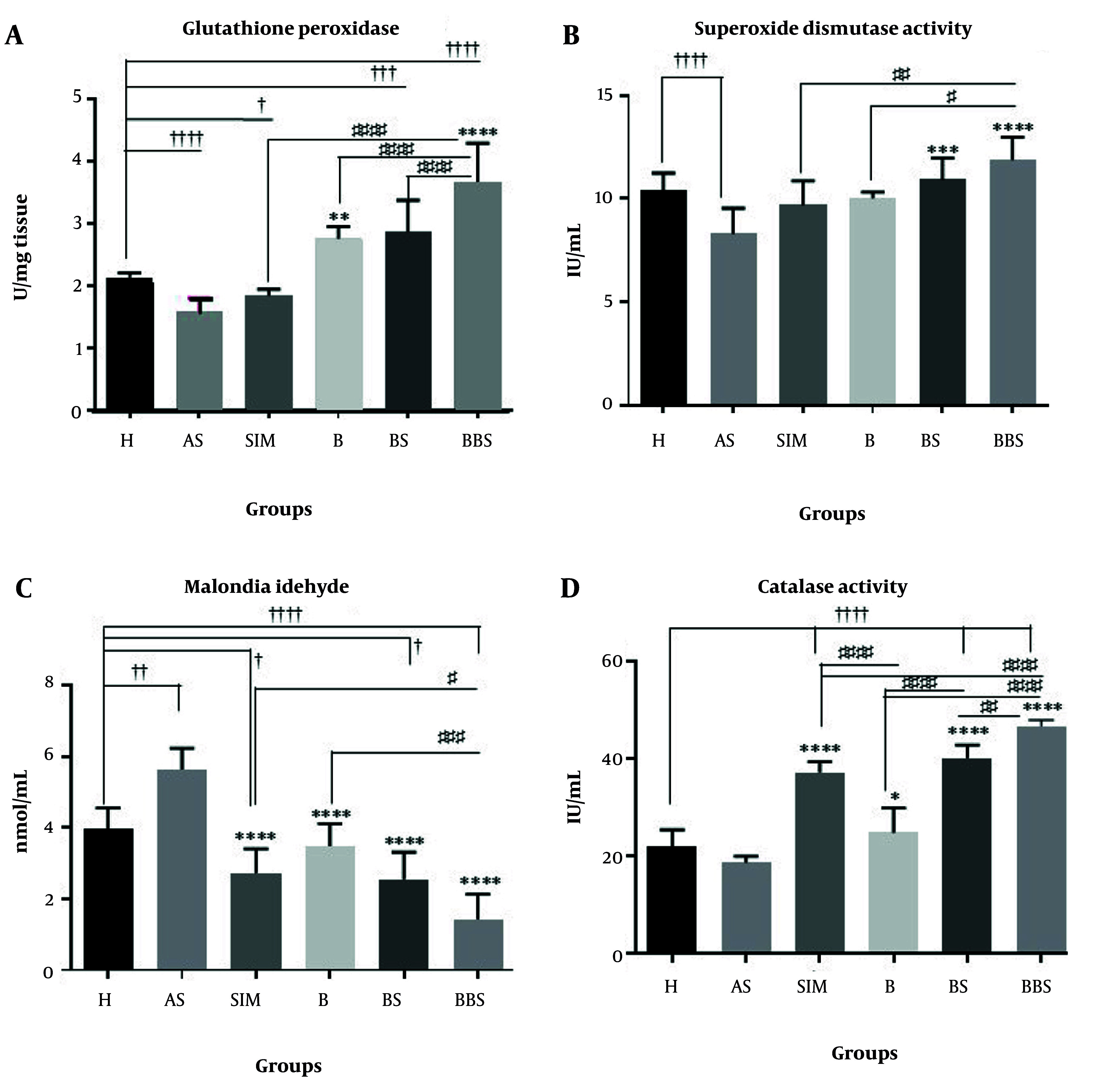
Effects of nanoemulsion berberine (BBR) on C57BL/6 J mice’s serum antioxidant status. A, glutathione peroxidase (GPX); B, superoxide dismutase (SOD) activity; C, malondialdehyde (MDA); and D, catalase (CAT) activity (Abbreviations: H, healthy; AS, atherogenic diet); SIM, AS and simvastatin (15 mg/kg); B, AS and BBR (30 mg/kg); BS, AS and nanoemulsion of black seed oil (10%); BBS, AS and BBR (1%) nanoemulsion of black seed oil (10%). Comparison of H group with AS, SIM, B, BS, and BBS groups † P < 0.05, †† P < 0.01, ††† P < 0.001, and †††† P < 0.0001; Comparison of AS group with H, SIM, B, BS, and BBS groups * P < 0.05, ** P < 0.01, *** P < 0.001, and **** P < 0.0001; Comparison of SIM, B, BS, and BBS groups together # P < 0.05, ## P < 0.01, ### P < 0.001, and #### P < 0.0001. Values are expressed as Mean ± SEM (n = 8).

The BBS group showed a significant increase in GPX activity, exceeding that of the H, SIM, B, and BS groups (P < 0.0001, Figure 4A). Additionally, BBS-treated subjects displayed elevated SOD levels compared to those in the SIM group (P < 0.01) and the B group (P < 0.05, Figure 4B). The BBS group exhibited a significant decrease in MDA levels, suggesting reduced oxidative stress. This reduction was statistically significant when compared to both the SIM group (P < 0.05) and the B group (P < 0.001, Figure 4C). Additionally, an analysis of CAT activity revealed that the BBS treatment significantly increased CAT levels. This increase was statistically significant when compared to the SIM group (P < 0.0001), the B group (P < 0.0001), and the BS group (P < 0.01) (Figure 4D). 

### 4.6. Aorta Histopathological Examination

**Figure 5.**
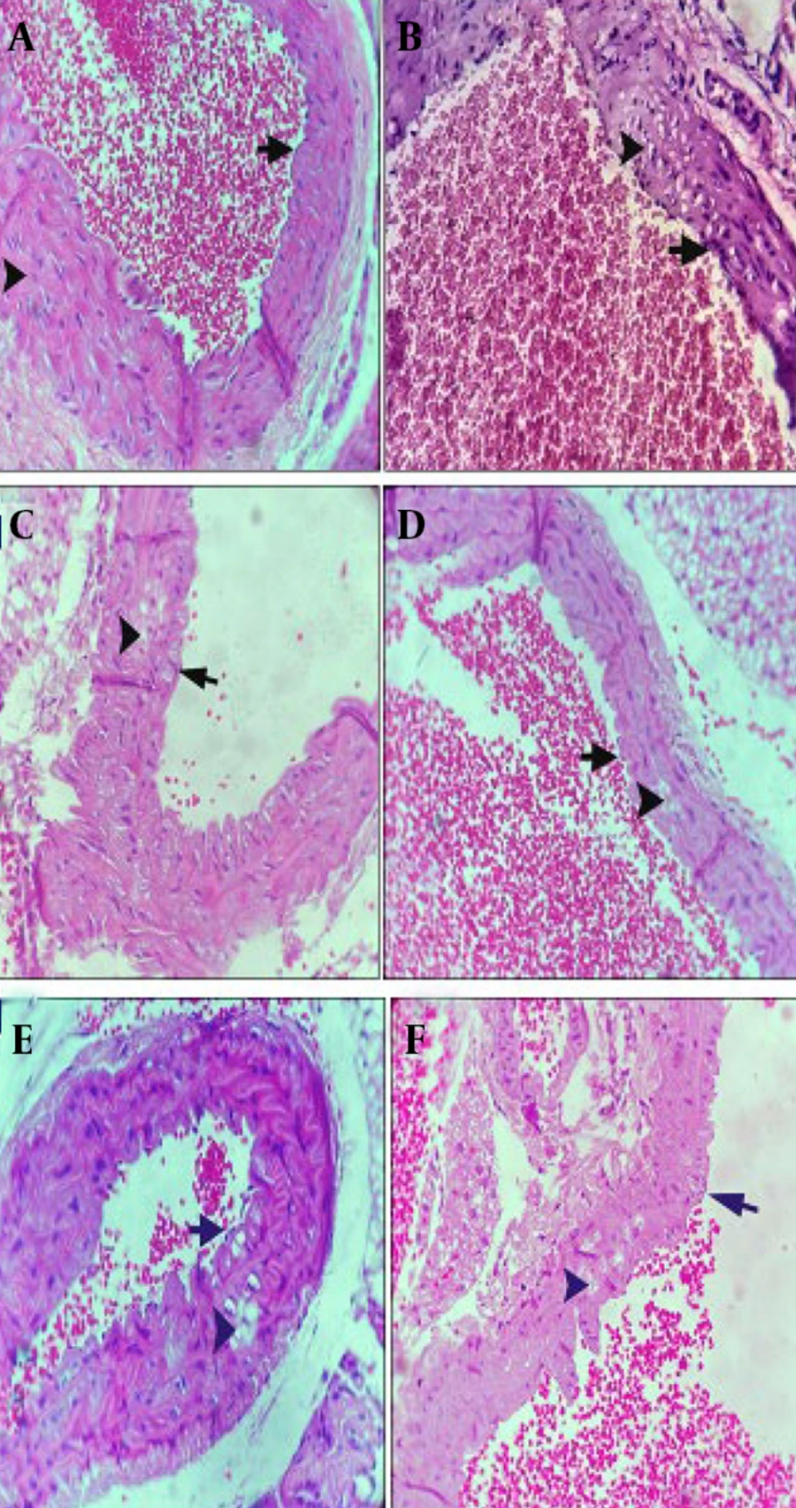
A, the middle layer of the vessel in the healthy control group is indicated by the arrowhead (400X); B, cholesterol plaques (arrowhead) and significant endothelial hyperplasia (arrow) in the AS group (400X). C, the SIM group consists of cholesterol plaques (arrowhead) and mild endothelial hyperplasia (arrow) (400X); D, small cholesterol plaques (arrowheads) in the medial layer of the vessel, healthy endothelium (arrow) (400X); E, in the BBS group, cholesterol plaques (arrowhead) and mild endothelial hyperplasia (arrow) (400X); F, in the BS group, significant cholesterol plaques (arrowhead) and mild endothelial hyperplasia (arrow) (400X).

Histological analysis revealed distinct differences between the H group and the AS group. The H group subjects exhibited normal aortic cross-sections, characterized by an intact medial layer and endothelium (Figure 5A). Conversely, the AS group presented with extensive cholesterol plaque formation and notable endothelial hyperplasia (Figure 5B). The SIM group was characterized by the presence of significant cholesterol plaques and mild endothelial hyperplasia (Figure 5C). In contrast, the B group displayed only a limited number of cholesterol plaques, alongside a healthy endothelium (Figure 5D). Comparatively, the BBS group exhibited distinct cholesterol plaques and mild hyperplasia (Figure 5E), whereas the BS group presented with substantial cholesterol plaques and slight hyperplasia (Figure 5F). Highly significant reduction in inflammation in the BBS group compared to the AS group (P < 0.0001).

**Figure 6.**
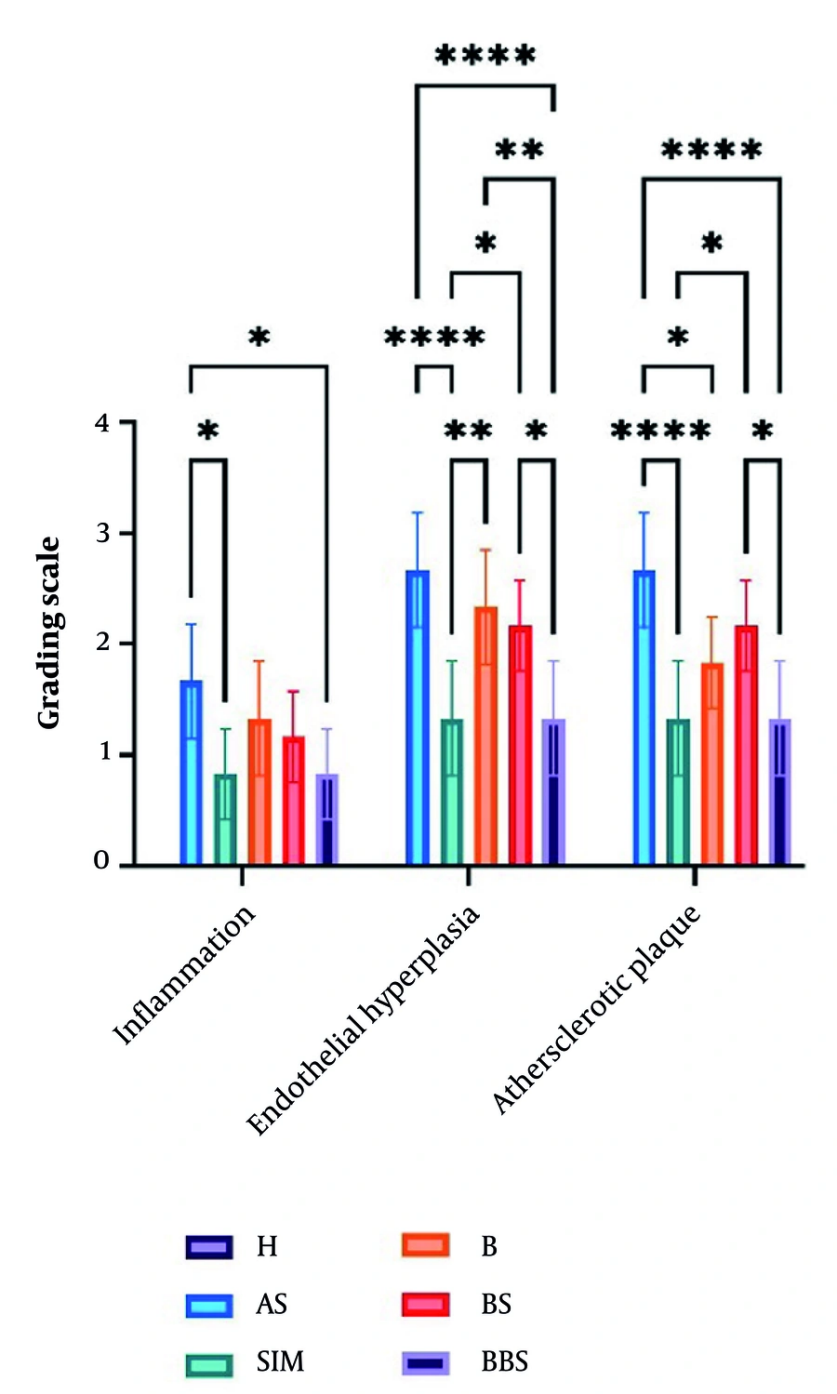
The atherosclerotic plaque grading used in the histology figure (Abbreviations: H, healthy; AS, atherogenic diet); SIM, AS and simvastatin (15 mg/kg); B, AS and BBR (30 mg/kg); BS, AS and nanoemulsion of black seed oil (10%); BBS, AS and BBR (1%) nanoemulsion of black seed oil (10%). Comparison of groups * P < 0.05, ** P < 0.01, and **** P < 0.0001.

The thickness of the intima and media layers was significantly greater in the AS group (P < 0.0001). Conversely, both SIM and BBS notably reduced endothelial hyperplasia compared to the AS group (P < 0.0001). The AS group exhibited a higher cholesterol plaque score (2.7), whereas the BBS and SIM groups had lower scores (1.33, P < 0.0001). Additionally, the AS group showed the highest infiltration of foam cells and macrophages (1.667). In contrast, the SIM and BBS groups demonstrated lower levels of inflammatory factor infiltration compared to the control group (P < 0.05, Figure 6).

## 5. Discussion

The integration of BBR with black seed oil nanoemulsion (BBS) shows significant therapeutic potential for managing dyslipidemia and oxidative stress, supported by detailed evaluations. The nanoemulsion demonstrated favorable physicochemical properties, including moderate polydispersity, stability (zeta potential: -18.4 mV), and a nanoscale particle size [dynamic light scattering (DLS): 250.4 nm; TEM: 117.32 nm]. The DLS reflects the hydrodynamic size, while TEM provides the actual core size, showing minor aggregation. Typically, DLS measurements are larger than those from TEM (23). These findings align with results reported in prior research (24, 25).

‎Recent scholarship highlights the promise of natural alternative therapies in addressing AS, particularly those exhibiting lipid-lowering, anti-inflammatory, and antioxidant effects comparable to those of statins (26). This escalating interest is primarily driven by the adverse reactions linked to conventional pharmacological interventions, including hemorrhage, gastrointestinal distress, and hepatic toxicity, which can compromise patients’ overall well-being. The 16-week high-fat diet induced consistent but modest plaque formation (primarily endothelial ‎hyperplasia with early foam cell infiltration), differing from advanced human lesions. This aligns ‎with established timelines for this model, where a significant plaque ‎burden typically requires more than 20 weeks.‎

The BBR, a dietary polyphenol and antioxidant, shows promise in inhibiting AS progression due to its anti-inflammatory properties. However, the exact mechanisms behind its cardiovascular benefits remain unclear, highlighting the need for further research. Studies also show that NS has lipid-modulating effects, largely due to thymoquinone (26). In animal models of hypercholesterolemia, thymoquinone significantly lowered total cholesterol, LDL, and TG while increasing HDL. Similar effects were observed with NS nanoemulsion powder and oil, resulting in a 15.5% reduction in serum cholesterol and a 22% reduction in TG. Clinical studies have shown that hypercholesterolemic patients who consumed one gram of black pepper daily for two months experienced significant reductions in total cholesterol, TG, and LDL, along with an increase in HDL (27).

A clinical trial involving 68 hyperlipidemic patients demonstrated that BBR significantly reduced total cholesterol (21.41%), TG (26.40%), and LDL (28.22%), comparable to the effects of statin therapy. The BBR was well-tolerated, with no adverse effects on renal function, and it improved hepatic function by upregulating LDL receptor expression (28).

Recent research indicates that BBR influences plasma cholesterol levels by activating adenosine monophosphate (AMP)-activated protein kinase, thereby inhibiting lipid synthesis in human hepatocytes. Furthermore, BBR protects against LDL oxidation and mitigates the cytotoxicity induced by oxidized LDL in endothelial cells. Nevertheless, BBR does not demonstrably impact HDL levels, which may restrict its comprehensive efficacy in lipid regulation (29).

Thymoquinone, a key compound in black seed, shows promise for preventing and treating AS due to its antioxidant properties. It inhibits reactive oxygen species (ROS), which contribute to oxidative stress and vascular inflammation. Research on hypercholesterolemic rabbits indicates that thymoquinone reduces oxidative stress, as shown by lower levels of MDA and protein carbonyls in serum and aortic tissue (30).

Research suggests that black seed has a positive impact on cholesterol and cardiovascular health. Supplementation with a black seed nanoemulsion resulted in reduced total and LDL cholesterol, along with improvements in TG, HDL, C-reactive protein (CRP), and antioxidant levels (31). Furthermore, the impact of black seed powder and oil on the advancement of AS was evaluated in rabbits maintained on a cholesterol-enriched diet. Over the course of eight weeks, black seed significantly improved lipid profiles and reduced aortic plaque formation. Additionally, black seed treatment resulted in a reduced intima-to-media ratio compared to the control group, suggesting a protective effect against aortic tissue damage induced by hypercholesterolemia (32). A study by Wang et al. found that feeding ApoE−/− mice a high-fat diet for 18 weeks led to the development of AS. An 8-week treatment with nicotinamide mononucleotide resulted in a 36% reduction in atherosclerotic plaque area, a 42% increase in early-stage lesions, and a 25% decrease in advanced-stage lesions. Additionally, there was a 48% smaller necrotic core and a 51% increase in collagen content, indicating improved plaque stability (33). A high-fat and high-carbohydrate diet (25% dietary fat, 1.25% cholesterol, and 5% sucrose) fed to male C57BL/6J mice for 16 weeks led to increased endothelial hyperplasia, cholesterol plaques, inflammation, and a larger necrotic core. In contrast, administering BBS for 8 weeks resulted in a 50% reduction in these factors. We recommend extending the diet period to 24 weeks for further observation of lesions (20).

The BBS demonstrates superior efficacy in improving serum lipid profiles compared to BBR or NS oil, significantly increasing HDL levels by 19.61% while reducing LDL, TG, and total cholesterol levels. It demonstrated strong antioxidant capacity, with GPX levels increasing 45% more than with simvastatin. Histopathological analyses confirmed its cardioprotective effects, showing reduced aortic plaque formation compared to the atherogenic control group. These findings suggest that BBS could be a potent multi-functional therapeutic agent for preventing AS and metabolic disorders, leveraging the lipid-lowering effects of BBR and the antioxidant properties of black seed oil.

## Data Availability

All data analyzed during this study, along with supporting information, are included in this published article. All data can be requested or viewed from the corresponding author upon request.
